# An in-person survey of the influence of the COVID-19 pandemic on physical function, functional capacity, cognitive function, and mental health among community-dwelling older adults in Japan from 2016 to 2022

**DOI:** 10.1186/s12877-024-05055-5

**Published:** 2024-05-24

**Authors:** Yuko Fukase, Naoto Kamide, Miki Sakamoto, Masataka Ando, Kanako Ichikura, Yoshitaka Shiba, Hirokuni Tagaya

**Affiliations:** 1 Kitasato University School of Allied Health Sciences, 1-15-1, Kitazato, Minami-ku, Sagamihara, Kanagawa 252-0373 Japan; 2 School of Health Sciences, Fukushima Medical University, 10-6 Sakaemachi, Fukushima City, Fukushima Japan

**Keywords:** COVID-19 pandemic, Community-dwelling older adults, Longitudinal survey, Lower-limb muscle strength, Depressive symptoms

## Abstract

**Background:**

The COVID-19 outbreak might have had several effects on older adults; however, much of the previous research only included self-report, cross-sectional, and online-survey data in the early stage of the pandemic. We conducted a face-to-face survey before and after the COVID-19 pandemic and investigated the influence of the pandemic on several functions to distinguish between changes due to aging and changes due to the pandemic using a linear mixed model.

**Methods:**

A total of 8 longitudinal surveys were conducted from 2016 to 2022. Physical function was assessed by weight, body mass index, body fat percentage, skeletal muscle mass index, calf circumference, grip strength, knee extension strength, the 5-times chair stand test, the timed up & go test and 5-m walking test. Functional capacity was measured using the Tokyo Metropolitan Institute of Gerontology index of competence, cognitive function was measured using the Trail Making Test - A, and mental health was measured using the Geriatric Depression Scale.

**Results:**

Of a total of 73 participants, 51 (69.9%) were female. The mean age at first participation was 71.82 years (SD = 4.64). The results of the linear mixed model showed that lower-limb muscle strength and body fat percentage and cognitive function changed significantly before and after the pandemic, while grip strength, functional capacity, and mental health did not.

**Conclusions:**

The changes in these functions between before and after the pandemic might be attributed to the diminished opportunities for the independent older individuals to go out and engage in activities. Although functional capacity did not change, lower-limb muscle strength is important for functional independence. This decline might influence the functional capacity of these individuals in the future.

**Supplementary Information:**

The online version contains supplementary material available at 10.1186/s12877-024-05055-5.

## Background

The COVID-19 outbreak caused people to restrict activities of daily living, which had several effects on overall function and health, such as anxiety, depressive symptoms, and a sense of loneliness [1–3]. Particularly in older adults, restricted social contact and mobility made them highly stressed [4], and the knowledge that they might be at a particularly high risk of developing COVID-19, suffering severe illness and dying as a result of it [5] accordingly resulted in negative effects on older adults’ quality of life, perceived health, and well-being [6].

On the other hand, Castell-Alcala et al. [7] showed that the influence due to the pandemic was small for the physical function, cognition, depressive mood, and quality of life in older adults. One reason for the differences in the results might be the survey methods and periods. Previous studies that involved online self-report surveys and cross-sectional surveys in the early stage of the pandemic showed that older adults felt stressed by the movement restricted in activities of daily living due to the pandemic [5, 8–11]. Conversely, research focusing on healthy/prefrail older adults utilizing longitudinal or face-to-face approaches suggested that the impact on physical activity or mental health during the pandemic was limited [6, 7, 12], whereas that conducted on individuals with disabilities demonstrated a decline in physical function [13]. According to Colucci et al. [6], more studies are needed to determine whether the secondary effects of confinement are long lasting or if quality of life, perceived health and well-being increased once seniors were able to resume participation in their usual activities. Accordingly, longitudinal face-to-face survey are needed for objective evaluation and to enable accurate conclusions.

In Japan, strict lockdowns have never been implemented, but mild lockdowns have been implemented intermittently because the pandemic has exhibited a repeated pattern of expansion and contraction. Previous research showed that the prevalence of depressive symptoms among the general population might have increased before the pandemic [8, 14, 15]. For older adults, flexibility and mobility deteriorated, and the prevalence of frailty increased during the pandemic [13, 16, 17]. Therefore, older adults in Japan have experienced long-term mobility restrictions due to the pandemic, and some of their physical function, functional capacity, cognitive function, and mental health might have been influenced by these restrictions. However, few studies have longitudinally investigated these functions starting before the pandemic and distinguished between changes caused by aging and those caused by the pandemic. The aim of the present study was to investigate the changes in various functions, that is physical function, functional capacity, cognitive function, and mental health, among older adults before and after the COVID-19 pandemic in Japan. The survey had two characteristics: (1) A face-to-face survey was conducted, which can measure not only functional capacity and mental health by self-report but also physical function and cognitive function. (2) The survey started in 2016 and investigated the changes in function before and after the pandemic, which could distinguish changes due to aging from changes due to the pandemic. The present study tried to distinguish between changes due to aging and changes due to the pandemic by a linear mixed model in which participants were included as a random factor and all survey times and survey times before and after the pandemic were included as fixed effects.

## Methods

### Procedure and participants

The participants in this study were recruited from the participants of health check-ups for geriatric syndrome held in Sagamihara City, Japan. Details of the health check-ups and the recruitment of participants are described elsewhere [18–22]. In summary, the participants were community-dwelling older people, and the health check-ups were held beginning in September 2016 with a 6-month interval. However, it could not be conducted between March 2020 and September 2021 because of the COVID-19 pandemic and was resumed in March 2022. Accordingly, the present study used data from 2016 to 2019 (waves 1 to 7) and March 2022 (wave 8) for analysis.

This study used the data of participants who (1) agreed to participate in the study; (2) were 65 years of age or older in 2016; (3) were not approved for the certification of support or care level by the long-term care insurance system in Japan; (4) participated after the pandemic (wave 8); and (5) participated more than 2 times before the pandemic (waves 1 to 7) to analyze changes from before the pandemic (waves 1 to 7).

### Measures

The present study collected age and sex as demographics and physical function, functional capacity, cognitive function, and mental health at all 8 timepoints (waves 1 to 8).

Anthropometrics and body composition parameters were evaluated through measurements of body weight, body mass index (BMI), body fat percentage (BFP), skeletal muscle mass index (SMI), and calf circumference (CC). Physical function was assessed by grip strength, knee extension strength (KES), 5-times chair stand test (CST), timed up & go test (TUG), and 5-m walking time.

Body fat and appendicular skeletal muscle mass were measured using a bioimpedance analysis method (InBody 430; InBody Japan Inc., Tokyo, Japan). Body fat percentage was calculated as body fat divided by body weight. SMI was calculated as appendicular skeletal muscle mass divided by the square of the height (m^2^). CC was measured as the maximum value of a calf of the nondominant limb using nonelastic tape. The grip strength was measured with the dominant-sided hand using a Smedley-type dynamometer (T.K.K.5401, TAKEI Scientific Instruments Co., Ltd., Niigata, Japan). KES was measured using a handheld dynamometer (µ-Tas F-1; Anima Inc., Tokyo, Japan) in a sitting position with the hip and knee joints flexed at 90 degrees, and isometric knee extension muscle strength at maximum effort was measured in the right leg. Regarding CST, a stopwatch was used to measure how long it took the participant to stand up from a sitting position and sit down with arms crossed in front of chest five times as quickly as possible. Regarding the TUG, the time to stand up from a chair without hand support, walk 3 m as quickly as possible, turn around, walk back, and sit down again was measured with a digital stopwatch (ALBA W072; Seiko Watch Corporation, Tokyo, Japan). Regarding 5-m walking time, the participants walked at a comfortable pace on a 9-m walkway, consisting of a measurement zone (5 m) and acceleration and deceleration zones (each 2 m); we measured the time taken to walk the 5-m length in the middle of the walkway using a digital stopwatch (ALBA W072; Seiko Watch Corporation, Tokyo, Japan).

Functional capacity was assessed by the Tokyo Metropolitan Institute of Gerontology index of competence (TMIG-IC), which has confirmed reliability and validity [23]. The TMIG-IC includes 13 items, and the score ranges from 0 to 13 points, with a higher score indicating higher capacity.

Cognitive function was evaluated using the Trail Making Test - A (TMT-A) [24], for which validity has been confirmed [25, 26]. Participants are asked to tap numbers ranging from 1 to 25 on a touchscreen as quickly as possible.

Mental health was measured with the five-item version of the Geriatric Depression Scale (GDS). The GDS is a self-administered questionnaire for assessing depressive symptoms among older adults, and its reliability and validity has been confirmed [27]. The total score ranges from 0 to 5 points, with a higher score indicating more severe depression.

#### Effect size

In the present study, it was difficult to control the sample sizes because it was a longitudinal study; accordingly, we calculated Cohen’s D (d) effect sizes for the t test, that is, 0.80 indicated a large effect size, 0.50 a medium effect size, and 0.20 a small effect size.

### Statistical analysis

The chi-square test and two-way analysis of variance (ANOVA) were used to compare the two groups at wave 7 according to inclusion status and sex. Among the participants in the present study, sociodemographic characteristics, physical function, functional capacity, cognitive function, and mental health status at first-time participation were compared according to sex using the chi-square test and two-sample t test.

To investigate changes in functions from waves 1 to 8 and before and after the pandemic, a linear mixed model with a random intercept was constructed. The fixed effects were sex, age at first-time participation, repeated scores from waves 1 to 8 (wave), and before (waves 1 to 7) and after (wave 8) the pandemic (COVID-19), and the random effect was individual differences. The wave was set as a fixed effect to examine the influence of changes over time across all survey periods (from September 2016 to September 2022). Furthermore, the “COVID-19” fixed effect was used to examine the influence of the COVID-19 pandemic. Regarding variables that changed before and after the pandemic (COVID-19), a linear mixed model with a random intercept and a slope model with the random effect were constructed and compared with the linear mixed model with a random intercept. Additionally, to examine an interaction, a linear mixed model including ‘sex*wave’ and ‘sex*COVID-19’ were constructed.

Estimated values and standard errors between Wave 1 (W1) and Wave 8 (W8) by sex are illustrated. The statistical significance level was set at *p* < 0.05, and Cohen’s d was used for the t test. Statistical analyses were carried out using IBM SPSS Statistics (Version 28) and the R programming language and environment (R version 4.2.2).

## Results

### Sociodemographic variables of the participants

**Fig. 1:**
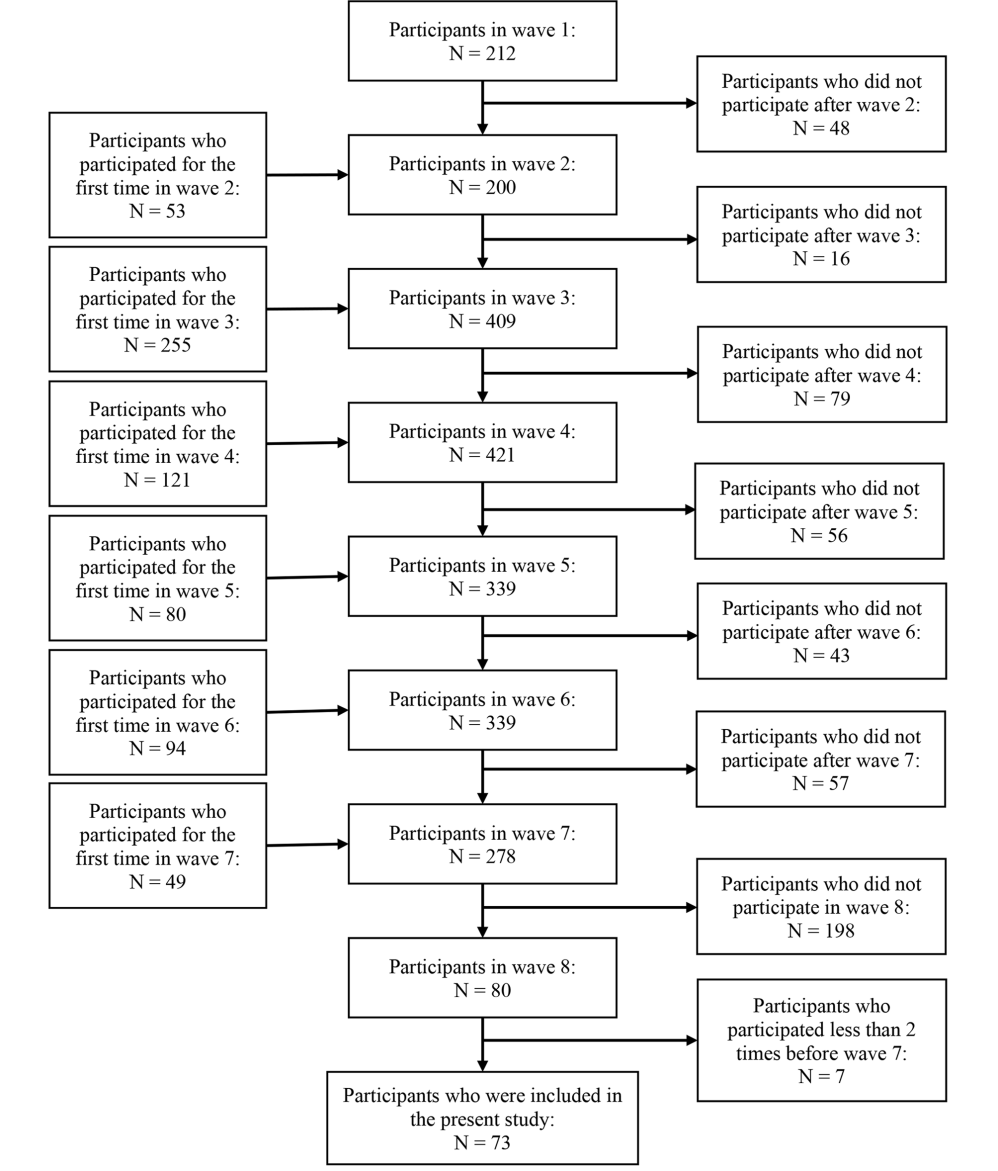
Flow chart of the present study

A detailed description of study participant inclusion is shown in Fig. 1. In wave 1, 212 participants participated. Between waves 2 to 7, new participants were included and some participants dropped out. In wave 7, 278 participants participated, 198 of whom did not participate in wave 8 (after the pandemic). At wave 8, 80 participants participated, 7 of whom were excluded from the analysis because they participated fewer than 2 times before wave 7. Ultimately, a total of 73 participants were analyzed.

Table 1 shows the results of the comparison between the 73 participants who were included in the present study and a total of 205 participants, including 182 participants who participated in wave 7 but did not participate in wave 8 and 23 participants who did not participate more than 2 times before the pandemic. There were no differences in the sex ratio or main group or interaction effects. Regarding TMIG-IC, there was a ceiling effect on female. Regarding GDS, all groups showed floor effects.

Regarding 73 participants who were included in the present study, all participants started participating at waves 1, 2, 3, or 4: the number of first-time participants was 19 (26.0%) at wave 1, 5 (6.8%) at wave 2, 29 (39.7%) at wave 3, and 20 (27.4%) at wave 4. Sociodemographic characteristics and function scores at initial participation are shown Table 2. Among the total participants, there were 22 (30.1%) males and 51 (69.9%) females, and there was a higher percentage of females. The mean age at first participation was 71.82 ± 4.64. Regarding function scores by sex, males were heavier, had a lower body fat percentage, had stronger grip strength, and had a higher skeletal muscle mass index than females, with large Cohen’s d values.

**Table 1:** A comparison between included and excluded participants at wave 7

	Included participants				Excluded participants					
	*N* = 73				*N* = 205				Statistics analysis	
	Male		Female		Male		Female		*p*-value	
	N/Mean	%/SD	N/Mean	%/SD	N/Mean	%/SD	N/Mean	%/SD	main effect of group	interaction
Sex, %	22	30.1%	51	69.9%	65	31.7%	140	68.3%	0.804 ^1)^	
Age (years)	74.14	4.61	73.80	4.51	74.59	5.34	72.67	4.46	0.624	0.256
Weight (kg)	60.21	5.82	51.07	9.40	62.90	7.59	51.42	7.82	0.199	0.322
BMI (kg/m^2^)	21.72	1.56	22.06	3.87	22.96	2.42	22.09	3.05	0.154	0.175
BFP (%)	20.06	3.74	28.17	7.20	21.57	5.24	28.62	6.85	0.301	0.575
GST (kg)	34.54	5.28	23.15	3.80	33.07	5.31	23.14	3.66	0.246	0.248
CC (cm)	36.10	1.72	33.99	2.89	36.52	2.48	34.39	2.66	0.293	0.977
KES (kg)	33.72	8.65	28.29	9.23	35.64	9.92	28.09	7.95	0.507	0.414
SMI (kg/m^2^)	7.31	0.45	6.07	0.77	7.61	0.69	6.12	0.60	0.065	0.199
CST (time)	6.70	1.70	5.87	1.22	6.83	1.94	6.28	1.38	0.236	0.537
TUG (sec)	5.65	0.87	5.64	0.79	5.54	0.98	5.72	0.84	0.905	0.449
5-m walking time										
Usual (sec)	3.55	0.43	3.52	0.62	3.57	0.62	3.42	0.45	0.605	0.427
Maximum (sec)	2.53	0.36	2.69	0.38	2.54	0.38	2.61	0.34	0.557	0.368
TMIG-IC	11.50	1.47	12.06	1.08	11.59	1.51	12.17	1.03	0.587	0.957
TMT-A (sec)	56.98	19.52	54.92	14.10	64.11	22.37	58.26	18.14	0.060	0.494
GDS	0.41	0.80	0.69	1.03	0.65	0.94	0.82	1.17	0.239	0.735

1) Chi-square test

BMI: Body mass index, BFP: Body fat percentage, GST: Grip strength, CC: Calf circumference, KES: Knee extension strength, SMI: Skeletal muscle mass index, CST: Chair stand test, TUG: Timed up & go test, TMIG-IC: Tokyo Metropolitan Institute of Gerontology index of competence, TMT-A: Trail Making Test - A, GDS: Geriatric Depression Scale-5

**Table 2:** Sociodemographic variables at initial participation (before the pandemic)

	Total			Male		Female		Statistics		
	*N* = 73			*N* = 22		*N* = 51				
	N/Mean		%/SD	N/Mean	%/SD	N/Mean	%/SD	χ^2^/t	*p*	D
Sex, %										
Male	22		30.1	-		-		11.52	< 0.001	-
Female	51		69.9	-		-				
Age (years)	71.82		4.64	72.14	4.78	71.69	4.62	0.38	0.707	0.10
Weight (kg)	53.72		10.14	59.80	7.51	51.10	10.06	3.63	< 0.001	0.93
BMI (kg/m^2^)	21.75		3.55	21.51	2.11	21.85	4.03	0.37	0.710	0.10
BFP (%)	25.12		7.79	19.69	4.66	27.47	7.73	5.30	< 0.001	1.12
GST (kg)	27.12		7.10	34.69	6.97	23.86	4.03	6.81	< 0.001	2.13
CC (cm)	34.30		3.05	35.20	2.13	33.93	3.30	1.63	0.108	0.42
KES (kg)	28.82		8.60	31.40	9.48	27.70	8.04	1.71	0.091	0.44
SMI (kg/m^2^)	6.43		0.90	7.29	0.50	6.06	0.76	6.91	< 0.001	1.76
CST (time)	6.19		1.41	6.75	1.89	5.95	1.09	1.85	0.075	0.58
TUG (sec)	5.63		0.81	5.72	0.93	5.58	0.76	0.67	0.503	0.17
5-m walking time										
Usual (sec)	3.45		0.46	3.39	0.26	3.47	0.52	0.90	0.372	0.18
Maximum (sec)		2.61	0.32	2.51	0.32	2.66	0.31	1.86	0.067	0.47
TMIG-IC	11.92		1.23	11.59	1.26	12.06	1.21	1.50	0.138	0.38
TMT-A (sec)	50.23		11.62	52.61	10.96	49.20	11.85	1.15	0.253	0.29
GDS	0.56		0.78	0.55	0.60	0.57	0.85	0.12	0.908	0.03

BMI: Body mass index, BFP: Body fat percentage, GST: Grip strength, CC: Calf circumference, KES: Knee extension strength, SMI: Skeletal muscle mass index, CST: Chair stand test, TUG: Timed up & go test, TMIG-IC: Tokyo Metropolitan Institute of Gerontology index of competence, TMT-A: Trail Making Test - A, GDS: Geriatric Depression Scale-5

### Change of variables from 2016 to 2022 and before and after the pandemic

The results of the linear mixed model with random intercept are shown Table 3. Values that did not change over the years (Wave) but changed before and after the pandemic (COVID-19) were calf circumference, knee extension strength, skeletal muscle mass index, chair stand test, 5-m usual walking time, 5-m maximum walking time, and TMT-A. The body fat percentage changed over the years (Wave) and before and after the pandemic (COVID-19), and the change level due to the pandemic was larger than that over the years. Values that changed over the years (Wave) and did not change before and after the pandemic (COVID-19) were BMI and grip strength. Values that did not change over the years (Wave) and before and after the pandemic (COVID-19) were weight, timed up & go test, TMIG-IC, and GDS.

The linear mixed model with a random intercept and the slope model with a random effect are shown in Supplementary Table 1. Regarding body fat percentage and TMT-A, although the *p* values did not change significantly before and after the pandemic (COVID-19), the estimated values were almost the same as those of the linear mixed model with a random intercept (Model 1). The other variables changed significantly before and after the pandemic (COVID-19).

The results of a linear mixed model including interaction terms, there was a significant interaction between sex and wave on calf circumference (Estimate = 0.19, SE = 0.06, *p* = 0.002), and the other variables had no significant interaction between sex and wave and between sex and COVID-19.

**Fig. 2:**
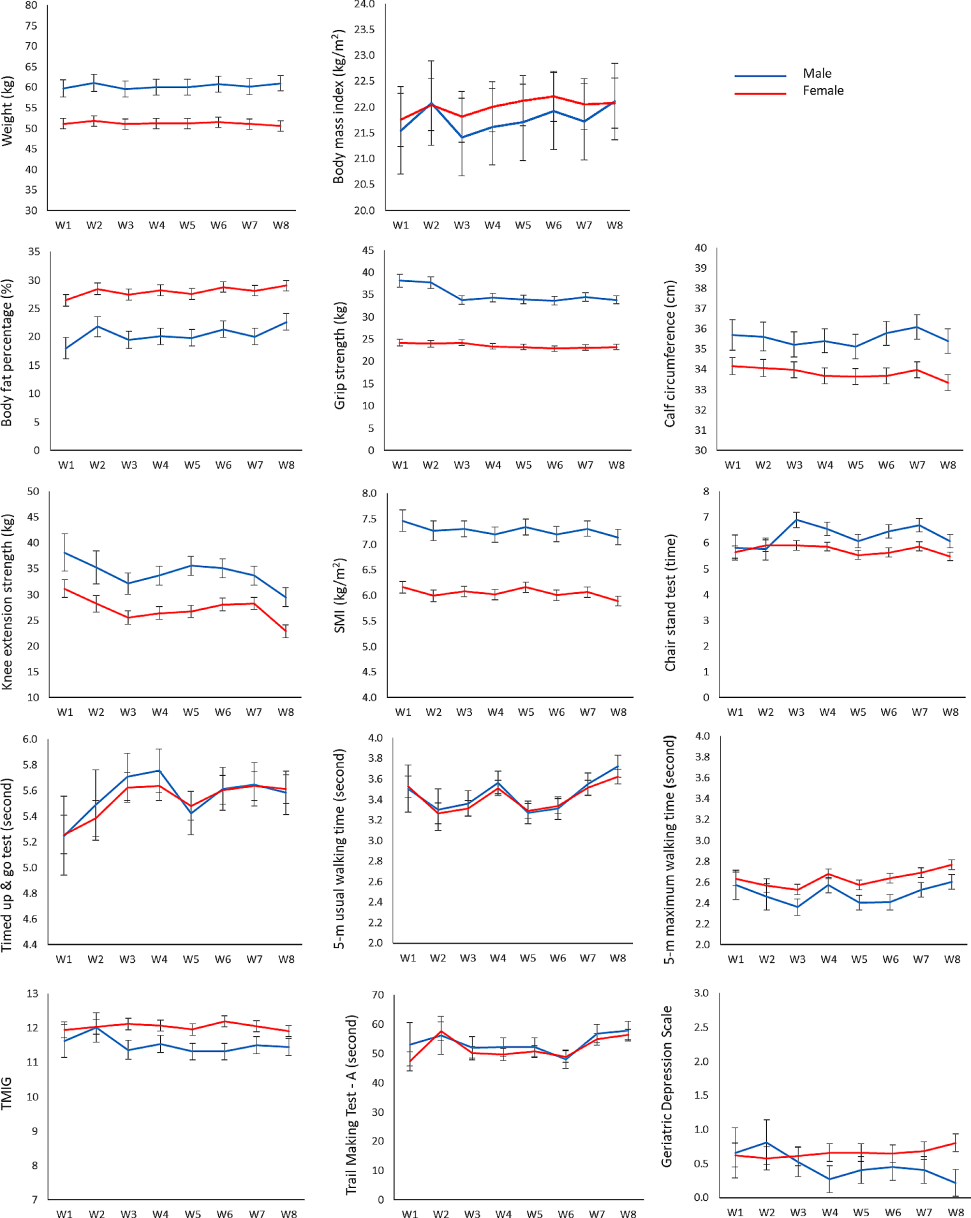
Estimate values and standard error between Wave 1 (W1) and Wave 8 (W8) W 1 to 7 were before the pandemic, and W8 was after the pandemic

Estimate values and standard errors between Wave 1 (W1) and Wave 8 (W8) by sex are shown in Fig. 2. In Fig. 2, W1 to W7 were regarded before the pandemic, and W8 was regarded after the pandemic.

**Table 3:** Estimate values of the linear mixed model. The estimated value of the ‘wave’ showed change over the years regardless of the pandemic, and ‘COVID-19’ showed change before and after the pandemic

		Estimate	SE	*p*				Estimate	SE	*p*
Weight	Intercept	83.04	15.72	< 0.001		CST	Intercept	0.51	1.87	0.785
	Sex	9.39	2.20	< 0.001			Sex	0.66	0.26	0.014
	Age	-0.44	0.22	0.047			Age	0.08	0.03	0.004
	Wave	0.02	0.05	0.763			Wave	0.00	0.02	0.841
	COVID-19	-0.27	0.28	0.343			COVID-19	-0.30	0.12	0.009
BMI	Intercept	27.74	6.25	< 0.001		TUG	Intercept	-1.17	0.97	0.231
	Sex	-0.25	0.87	0.778			Sex	-0.02	0.14	0.906
	Age	-0.08	0.09	0.343			Age	0.09	0.01	< 0.001
	Wave	0.05	0.02	0.015			Wave	0.02	0.01	0.091
	COVID-19	-0.02	0.11	0.837			COVID-19	-0.05	0.07	0.455
BFP	Intercept	32.03	11.89	0.009		5-m usual walking time	Intercept	1.08	0.70	0.127
	Sex	-7.56	1.66	< 0.001			Sex	0.02	0.10	0.857
	Age	-0.08	0.17	0.628			Age	0.03	0.01	0.004
	Wave	0.18	0.07	0.008			Wave	0.01	0.01	0.469
	COVID-19	0.85	0.35	0.018			COVID-19	0.22	0.06	< 0.001
GST	Intercept	49.58	7.05	< 0.001		5-m maximum walking time	Intercept	0.25	0.44	0.572
	Sex	11.04	0.98	< 0.001			Sex	-0.18	0.06	0.005
	Age	-0.36	0.10	0.001			Age	0.03	0.01	< 0.001
	Wave	-0.23	0.06	< 0.001			Wave	0.01	0.01	0.054
	COVID-19	0.50	0.31	0.106			COVID-19	0.10	0.03	0.004
CC	Intercept	46.21	4.76	< 0.001		TMIG-IC	Intercept	11.77	1.76	< 0.001
	Sex	1.87	0.66	0.006			Sex	-0.61	0.25	0.015
	Age	-0.17	0.07	0.013			Age	0.01	0.02	0.825
	Wave	0.01	0.03	0.578			Wave	0.00	0.02	0.966
	COVID-19	-0.42	0.13	0.002			COVID-19	-0.10	0.12	0.376
KES	Intercept	65.54	12.76	< 0.001		TMT-A	Intercept	-22.49	18.25	0.225
	Sex	7.10	1.78	< 0.001			Sex	0.83	2.54	0.746
	Age	-0.47	0.18	0.010			Age	0.95	0.25	0.001
	Wave	0.09	0.17	0.614			Wave	0.30	0.32	0.362
	COVID-19	-4.85	0.88	< 0.001			COVID-19	4.23	1.69	0.013
SMI	Intercept	8.64	1.21	< 0.001		GDS	Intercept	0.99	1.44	0.492
	Sex	1.23	0.17	< 0.001			Sex	-0.28	0.20	0.161
	Age	-0.03	0.02	0.051			Age	-0.01	0.02	0.787
	Wave	0.00	0.01	0.598			Wave	0.01	0.02	0.713
	COVID-19	-0.15	0.04	< 0.001			COVID-19	0.03	0.09	0.716

BMI: Body mass index, BFP: Body fat percentage, GST: Grip strength, CC: Calf circumference, KES: Knee extension strength, SMI: Skeletal muscle mass index, CST: Chair stand test, TUG: Timed up & go test, TMIG-IC: Tokyo Metropolitan Institute of Gerontology index of competence, TMT-A: Trail Making Test – A, GDS: Geriatric Depression Scale-5

## Discussion

The present study investigated the influence of the COVID-19 pandemic on multiple functions among older adults. First, the participants who were included in the present study were not different from the participants who were excluded from the analysis. Participants might have good functional capacity and mental health based on TMIG-IC and GDS values.

The results of the linear mixed model showed that calf circumference, knee extension strength, skeletal muscle mass index, chair stand test, usual or maximum walking time of physical function and cognitive function were influenced significantly by the pandemic and not influenced by aging (wave). Additionally, body fat percentage increased before and after the pandemic more than over the years, and grip strength, functional capacity, and mental health did not change before and after the pandemic.

The reason for the decrease in lower limb muscle strength among our participants who were independent during the pandemic may be attributed to reduced walking compared to prefrail adults, whose activity levels were less affected. Previous research indicated that scores on the Short Physical Performance Battery (including the chair stand test, balance tests, and gait speed test) did not decline among prefrail older adults during the COVID-19 pandemic [7]. One potential explanation for the disparity between our findings and previous research could be the characteristics of the participants. Our study focused on independent older adults who had sufficient lower-limb muscle strength and freedom of movement before the pandemic. Due to the COVID-19 pandemic, opportunities for walking may have decreased, leading to a decline in lower-limb muscle strength among independent older adults. Conversely, prefrail older adults might have already faced limitations in their activities prior to the pandemic, thus experiencing less significant changes in their activity levels.

Additionally, grip strength declined due to aging (wave) but did not decline before and after the pandemic in the present research. There are many reports that lower-limb muscle strength declines more easily than upper-limb muscle strength [28, 29], which suggests that the upper-limb muscle tends to be used more often than the lower-limb muscle even though their movements are restricted.

The impact of the mobility restrictions due to the pandemic may extend to loss of functional independence via lower-limb muscle weakening [30]. A decline in lower-limb muscle strength causes disability in walking speed, balance ability, and independence in daily life [31, 32]. Guidelines for the prevention of falls in older persons indicate that one of the strongest triggers for the risk of falling is a decline in lower-body strength [33]. Although in the present research, the level of functional capacity measured by TMIG-IC did not change before and after the pandemic, there is a possibility that the decline in lower-limb muscle strength will lead to a disability in physical function and functional capacity in the future.

The present study showed that cognitive function, which was measured by TMT-A, did not decline due to aging (wave) but declined due to the diminished opportunities to go out and engage in activities. TMT-A especially measures visual scanning, graphomotor speed, and visuomotor processing speed [34]. Woods et al. [35] suggested that the reason older adults exhibit lower TMT performances than younger adults are because of the decrease in travel time with age. However, Salthouse et al. [36] pointed out that age was not directly related to performance, but perceptual speed was more related to performance. According to those suggestions and the results of the present study, it is thought that cognitive function determined by the TMT-A declines in the long term, but the decline might not appear over several years, and before and after the pandemic, cognitive function might decline, notably that of processing speed, such as visuomotor processing speed and perceptual speed. The impact on the decline in processing speed needs to be investigated by a follow-up survey.

In the present study, depressive symptoms were not influenced by age, survey time, or diminished opportunities for going out. Systematic reviews have suggested that the pandemic has influenced mental health among the general population; however, the influence of the pandemic on the mental health of older adults might be small [37, 38]. In addition to the stressful life events, lifestyle, and coping strategies examined in the present study, genetic and physiological factors also influenced depressive symptoms [39]. The participants in the present study might have been energetic enough to voluntarily participate in the health check-ups and might have maintained their health well enough to continue to participate. Accordingly, there is a possibility that older adults at high risk for depression were excluded from the present study. Additionally, depressive symptoms might have temporarily worsened during the period when the survey could not be conducted due to the pandemic. Therefore, studies that include older adults at high risk for depression and studies conducted during the pandemic are needed.

This study revealed that diminished opportunities to go out and engage in activities affected the results of functional tests, such as knee extension strength and skeletal muscle mass, that can only be administered via face-to-face physical testing. Regarding the methodological approach, many previous studies on older adults that were conducted during the pandemic measured the participants’ function by a self-report questionnaire, which was a suitable method during the pandemic because it can be conducted online and by mail. However, it is usually difficult to detect a subtle decline in a subject’s muscle strength or muscle mass using those questionnaire-based surveys. The present study showed that only face-to-face physical tests can accurately reveal functional decline among older adults. Moreover, there is a need not only for easy methods of assessing performance levels, such as the chair stand test and walking speed, which presumably indicate muscle mass and muscle strength changes, but also for measures of functional levels that can be measured directly by a medical professional, including muscle strength (especially knee extension strength, which requires more skill to assess than measures such as grip strength) and muscle mass.

### Limitations

The present study analyzed participants who participated in health check-ups and kept participating before and after the pandemic; accordingly, participants tended to be independent in activities of daily living and might be health-conscious people. Therefore, the results cannot be generalized for those with prefrailty and frailty. The survey could not be conducted between 2020 and 2021 because of the COVID-19 pandemic; therefore, the present study could discuss these functions before and after the pandemic but could not discuss them during the pandemic. In addition, the limitation of this study include that interactions terms, such as sex and the survey times could not be considered. The interactions need to be examined by limiting the factors. The measurements had some limitations: functional capacity and mental health were measured only self-reported methods. It is necessary to measure these functions by multiple methods, such as structured interview by medical profession.

## Conclusions

The present study showed that lower-limb muscle strength, cognitive function, and body fat percentage changed before and after the COVID-19 pandemic. Lower-limb muscle strength did not decline over the years but declined before and after the pandemic, and upper-limb muscle strength was not affected by the pandemic. It is suggested that opportunities for walking for as long as they used to do were lost due to the pandemic especially for independent older adults, which might explain the lower-limb muscle strength decline and body fat percentage increase observed in this study. Additionally, cognitive function, especially processing speed, might decline due to the diminished opportunities to go out. There is concern that the decline in lower-limb muscle strength and cognitive function will influence their functional capacity in the future.

### Electronic supplementary material

Below is the link to the electronic supplementary material.

Supplementary Material 1

## Data Availability

The datasets generated during the current study are available in the openICPSR database, 10.3886/E188621V1.

## Abbreviations

BMI: body mass index
BFP: body fat percentage
SMI: skeletal muscle mass index
CC: calf circumference
GST: grip strength
KES: knee extension strength
CST: 5-times chair stand test
TUG: timed up & go test
TMIG-IC: Tokyo Metropolitan Institute of Gerontology index of competence
TMT-A: Trail Making Test - A
GDS: the five-item version of the Geriatric Depression Scale
ANOVA: analysis of variance
