# Supplementary material for: An in-person survey of the influence of the COVID-19 pandemic on physical function, functional capacity, cognitive function, and mental health among community-dwelling older adults in Japan from 2016 to 2022
Source: BMC Geriatr. 2024 May 24;24:457. doi: 10.1186/s12877-024-05055-5 (PMC11127291; doi:10.1186/s12877-024-05055-5)
Supplement: Supplementary file 1 — Supplementary Material 1 [file 12877_2024_5055_MOESM1_ESM.docx]

Supplementary Table 1. The linear mixed model with random intercept (Model 1) and with random intercept and slope model as random effect (Model 2).

|  |  |  | Model 1 ^a)^ |  |  |  | Model 2 ^b)^ |  |
| --- | --- | --- | --- | --- | --- | --- | --- | --- |
|  |  | Estimate | SE | p |  | Estimate | SE | p |
| BFP | Intercept | 32.03 | 11.89 | 0.009 |  | 24.85 | 12.00 | 0.042 |
|  | Sex | -7.56 | 1.66 | < 0.001 |  | -7.68 | 1.66 | < 0.001 |
|  | Age | -0.08 | 0.17 | 0.628 |  | -0.09 | 0.17 | 0.596 |
|  | Wave | 0.18 | 0.07 | 0.008 |  | 0.20 | 0.06 | 0.001 |
|  | COVID-19 | 0.85 | 0.35 | 0.018 |  | 0.82 | 0.47 | 0.084 |
| CC | Intercept | 46.21 | 4.76 | < 0.001 |  | 47.00 | 4.70 | < 0.001 |
|  | Sex | 1.87 | 0.66 | 0.006 |  | 2.03 | 0.65 | 0.003 |
|  | Age | -0.17 | 0.07 | 0.013 |  | -0.15 | 0.06 | 0.022 |
|  | Wave | 0.01 | 0.03 | 0.578 |  | 0.02 | 0.02 | 0.513 |
|  | COVID-19 | -0.42 | 0.13 | 0.002 |  | -0.42 | 0.14 | 0.003 |
| KES | Intercept | 65.54 | 12.76 | < 0.001 |  | 70.99 | 12.14 | < 0.001 |
|  | Sex | 7.10 | 1.78 | < 0.001 |  | 6.95 | 1.67 | < 0.001 |
|  | Age | -0.47 | 0.18 | 0.010 |  | -0.45 | 0.17 | 0.009 |
|  | Wave | 0.09 | 0.17 | 0.614 |  | 0.10 | 0.17 | 0.565 |
|  | COVID-19 | -4.85 | 0.88 | < 0.001 |  | -4.91 | 0.89 | < 0.001 |
| SMI | Intercept | 8.64 | 1.21 | < 0.001 |  | 9.98 | 1.17 | < 0.001 |
|  | Sex | 1.23 | 0.17 | < 0.001 |  | 1.26 | 0.16 | < 0.001 |
|  | Age | -0.03 | 0.02 | 0.051 |  | -0.03 | 0.02 | 0.034 |
|  | Wave | 0.00 | 0.01 | 0.598 |  | 0.00 | 0.01 | 0.628 |
|  | COVID-19 | -0.15 | 0.04 | < 0.001 |  | -0.15 | 0.04 | < 0.001 |
| CST | Intercept | 0.51 | 1.87 | 0.785 |  | 1.39 | 1.80 | 0.442 |
|  | Sex | 0.66 | 0.26 | 0.014 |  | 0.62 | 0.25 | 0.015 |
|  | Age | 0.08 | 0.03 | 0.004 |  | 0.07 | 0.02 | 0.004 |
|  | Wave | 0.00 | 0.02 | 0.841 |  | -0.01 | 0.02 | 0.798 |
|  | COVID-19 | -0.30 | 0.12 | 0.009 |  | -0.30 | 0.12 | 0.012 |
| 5-m usual walking time | Intercept | 1.08 | 0.70 | 0.127 |  | 1.14 | 0.70 | 0.111 |
|  | Sex | 0.02 | 0.10 | 0.857 |  | 0.01 | 0.10 | 0.892 |
|  | Age | 0.03 | 0.01 | 0.004 |  | 0.03 | 0.01 | 0.005 |
|  | Wave | 0.01 | 0.01 | 0.469 |  | 0.01 | 0.01 | 0.464 |
|  | COVID-19 | 0.22 | 0.06 | < 0.001 |  | 0.22 | 0.06 | < 0.001 |

Supply Table 1. Continued.

|  |  |  | Model 1 ^a)^ |  |  |  | Model 2 ^b)^ |  |
| --- | --- | --- | --- | --- | --- | --- | --- | --- |
|  |  | Estimate | SE | p |  | Estimate | SE | p |
| 5-m maximum walking time | Intercept | 0.25 | 0.44 | 0.572 |  | 0.09 | 0.44 | 0.831 |
|  | Sex | -0.18 | 0.06 | 0.005 |  | -0.18 | 0.06 | 0.004 |
|  | Age | 0.03 | 0.01 | < 0.001 |  | 0.03 | 0.01 | < 0.001 |
|  | Wave | 0.01 | 0.01 | 0.054 |  | 0.01 | 0.01 | 0.052 |
|  | COVID-19 | 0.10 | 0.03 | 0.004 |  | 0.10 | 0.04 | 0.007 |
| TMT-A | Intercept | -22.49 | 18.25 | 0.225 |  | -19.33 | 16.15 | 0.235 |
|  | Sex | 0.83 | 2.54 | 0.746 |  | 0.82 | 2.21 | 0.713 |
|  | Age | 0.95 | 0.25 | 0.001 |  | 0.92 | 0.22 | < 0.001 |
|  | Wave | 0.30 | 0.32 | 0.362 |  | 0.29 | 0.35 | 0.407 |
|  | COVID-19 | 4.23 | 1.69 | 0.013 |  | 4.24 | 2.16 | 0.052 |

a) Model 1: The linear mixed model with random intercept.

b) Model 2: The linear mixed model with random intercept and slope model as random effect.

BFP: Body fat percentage, CC: Calf circumference, KES: Knee extension strength, SMI: Skeletal muscle mass index, CST: Chair stand test, TMT-A: Trail Making Test – A.
